# Mindfulness mediates the relationship between positive parenting and aggression, depression, and suicidal ideation: A longitudinal study in middle school students

**DOI:** 10.3389/fpsyg.2022.1007983

**Published:** 2022-11-03

**Authors:** Yanhua Su, Wenyan Sun, Yiqun Gan, Qian Zhu, Guoyan Liu, Linhu Hui, Hui Tang, Zhijun Liu

**Affiliations:** ^1^Center for Mental Health Research in School of Management, Zunyi Medical University, Zunyi, China; ^2^Children’s Hospital Affiliated to Zhengzhou University, Zhengzhou, China; ^3^School of Psychological and Cognitive Science, Peking University, Beijing, China; ^4^School of Medical Information Engineering, Zunyi Medical University, Zunyi, China; ^5^School of Education, Zhaoqing University, Zhaoqing, China

**Keywords:** positive parenting, suicidal ideation, aggression, depression, mindfulness

## Abstract

Previous research has indicated that parenting factors affect the risk of maladaptive psychological outcomes (e.g., aggression, depression, or suicidal ideation), and that positive parenting is a prospective risk factor for maladaptive psychological outcomes. However, the mechanisms underlying the relationships between positive parenting, mindfulness, and maladaptive psychological outcomes remain unknown, as do the processes that mediate the effect of positive parenting on maladaptive psychological outcomes in adolescents. The objective of the present study was to investigate the longitudinal relationship between positive parenting, mindfulness, and maladaptive psychological outcomes in middle school students, as well as the mediating effect of mindfulness in the relationships between positive parenting and depression, aggression, and suicidal ideation. In this study, 386 middle school children (aged 12–16) were tested three times over a period of 6 months. Positive parenting was assessed at Time 1, mindfulness at Time 2, and depression, aggression, and suicidal ideation at Time 3. Using structural equation modeling, positive parenting was revealed to be longitudinally associated with mindfulness and negatively associated with maladaptive psychological outcomes. More crucially, mindfulness mediated the relationship between positive parenting and maladaptive psychological outcomes. This research provides important insights into how to effectively decrease adolescent maladaptive psychological outcomes and highlights the importance of teaching mindfulness to youths.

## Introduction

Adolescence is a significant time of growth during which mental health issues can emerge (Polanczyk et al., 2015). The physiological and psychological changes that occur during puberty can trigger major public health problems, such as suicidal behaviors (suicidal ideation or suicide attempts; Xiao et al., 2019; Xu et al., 2022), depression, and aggression (Achenbach, 1991; Achenbach and Edelbrock, 1991; Reitz et al., 2005; Graber, 2013; Archana and Kumar, 2022). Worldwide, suicide is a serious public health problem and a leading cause of death and disability, especially among adolescents (McLoughlin et al., 2015; Xiao et al., 2019; Xu et al., 2022). Suicidal ideation, a key step in the suicide process, is a significant risk factor for youth suicide (Cluver et al., 2015; Franklin et al., 2017). Suicidal ideation is prevalent among Chinese adolescents, with rates ranging between 15.7% (*N* = 54,640) and 18.2% (*N* = 20, 475) (Guo et al., 2019; Xiao et al., 2019; Xu et al., 2022). Additionally, depression is significant global public health issue (Ferrari et al., 2013), with incidence rates ranging from 2 to 7% in children and adolescents (Alsaad et al., 2018). Depression, which has been associated with decreased academic performance, family problems, drug abuse, and decreased life expectancy (Murray et al., 2015), is frequently accompanied by a high suicide risk (Murray et al., 2015; Apter and Gvion, 2016). Aggression is defined as behavior intended to hurt oneself or others (Siever, 2008), and is another public health issue, with a frequency of 25% in China (Peng et al., 2022). Several studies have noted that adolescents with more aggressive behavioral tendencies and patterns are more likely to have poorer physical and mental health, scholastic performance, personality development, and social adaption than others (Gini and Pozzoli, 2013; Gini et al., 2013).

Due to the prevalence of aggression, depression, and suicidal ideation in adolescents, research on maladaptive psychological outcomes in the teenage population has frequently concentrated on identifying their causes (Dumais et al., 2005; Augsberger et al., 2018; Lee and Ham, 2018). Increasing evidence has demonstrated that positive parenting can contribute to the prevention of these problems in Western cultural contexts (Boeldt et al., 2012; Smokowski et al., 2015). Despite the extensive reports from Western countries that positive parenting is associated with improved child and adolescent psychosocial and developmental outcomes, the majority of these studies have tested bivariate relationships rather than mediational models, and have not used longitudinal data. According to Social Emotional Learning (SEL) theory (Tolan et al., 2016), positive parenting can enhance children’s social and emotional skills, and indirectly foster the development of social-emotional competencies, such as mindfulness, in adolescents. Thus, with a focus on prevention, we used longitudinal methods to investigate the mediating effect of adolescents’ mindfulness in the associations between positive parenting, depression, aggression, and suicidal ideation.

While the relationships between positive parenting and depression, aggression, and suicidal ideation have been well-documented in Western countries (Chen and Raine, 2018; Kingsbury et al., 2020), relatively little research has been conducted on this topic in a Chinese environment. A Chinese adage states that “Beating and reprimanding are the signs of love.” This demonstrates that, in the context of traditional Chinese culture, controlling or autonomy-restrictive parental practices (e.g., harsh parenting behaviors) are more normative in China (Liu et al., 2021) than in Western contexts. Indeed, these practices are still adopted by approximately 50% of Chinese parents (Wang and Liu, 2014) and thus seem normal and appropriate in the eyes of Chinese adolescents (Chao, 1994; Cheung and Pomerantz, 2011). Shek (2007) found that although Chinese parents are accustomed to scolding their children, screaming at them, and even punishing them for failing to achieve their expectations, Chinese adolescents do not necessary perceive these parenting practices as harsh. It is still unknown whether the positive parenting in the context of Chinese culture will have the same effect as that in Western countries. Given these cultural understandings, it is worth investigating further the relationship between positive parenting and maladaptive psychological outcomes in a Chinese context.

### The importance of positive parenting in preventing aggression, depression, and suicidal ideation

Many studies have shown that parental practices are crucial contributors to the emergence and maintenance of adolescents’ mental and behavioral problems, including depression (Schwartz et al., 2014), aggression (Hentges et al., 2018), and suicide (Borkowski et al., 2001; Maccoby, 2001; Fotti et al., 2006). According to the family system theory, families are not just groups of people living together, but rather multilevel systems in which the parents play essential roles as both leaders and implementers (Bowen, 1974). Therefore, everyone, especially adolescents, is affected by any change in any element of the system. Following this, then, positive parenting behaviors have been hypothesized to decrease youth depression, aggression, and suicidal ideation (You and Lim, 2015; Kingsbury et al., 2020).

Positive parenting refers to warm, supportive parental behaviors that demonstrate acceptance, praise, and responsibility, including but not limited to nurturing, instructing, listening, expressing approbation, and providing emotional support (Russell, 1996; Chronis et al., 2007; Boeldt et al., 2012; Kingsbury et al., 2020). In addition, any positive component that stems from specific parenting behaviors and beliefs and enters into the parenting environment can be considered a part of positive parenting (Roskam, 2015). There is considerable evidence that positive parenting is a key buffer against the emergence of depression, aggression (Pinquart, 2017a,b; Nieto-Casado et al., 2022), and suicidal ideation (Kingsbury et al., 2020). Smokowski et al. (2015) found that positive parenting was negatively associated with depression. In another study, Jakobsen et al. (2012) revealed that adolescents who reported parental support had less severe anxiety and depressive symptoms. A significant association between positive parenting and aggressive behavior has also been reported (Chen and Raine, 2018). In particular, aggressive behavior was more common in children who had been exposed to negative parenting styles than in children who had been exposed to positive parenting styles (Lei et al., 2018). More importantly, a substantive body of empirical research has asserted that a supportive family environment could defend against the stresses that lead to suicidal ideation and actions in young people (Zhang and Jin, 1996; Lai and McBride-Chang, 2001). Indeed, family support and connectedness has been shown to reduce the likelihood of suicidal thoughts and behaviors (Sharaf et al., 2009; Purcell et al., 2012).

According to several cross-sectional studies, the presence of parental warmth (Machell et al., 2016), sufficient autonomy support (Shpigel et al., 2012), and efficient communication (Mark et al., 2013) are protective factors against the formation of suicidal thoughts in adolescents. In a more recent longitudinal study, Kingsbury et al. (2020) found that positive parenting was protective against depression, anxiety, and physical and social aggression. Taken together, the evidence points to a strong negative correlation between positive parenting and maladaptive psychological outcomes such as depression, aggression, and suicidal ideation (Purcell et al., 2012; Smokowski et al., 2015; Pinquart, 2017a,b; Chen and Raine, 2018; Nair et al., 2020).

### The mediating role of mindfulness

The SEL theory is a model of positive development (Tolan et al., 2016) that describes the process of helping people acquire the “capacity to understand, control, and express one’s social and emotional aspects of one’s life” (Elias et al., 1997). The SEL model (Tolan et al., 2016) holds that positive parenting can cultivate social and emotional abilities that directly or indirectly contribute to competency development in adolescents by improving the environments that support the development of these abilities in adolescents. Indeed, some studies have shown that positive parenting plays a significant part in the healthy development of adolescents because it supports the acquisition of skills, promotes self-acceptance, boosts self-esteem, and facilitates the formation of secure attachments (Smalls, 2009; Elmore and Gaylord-Harden, 2013). Other studies have shown that children who have secure, stable relationships with their caregivers are also more likely to acquire a greater capacity for mindfulness (Shaver et al., 2007; Pepping et al., 2015; Lucas-Thompson et al., 2021; Nieto-Casado et al., 2022). Parent–child interactions that are marked by warmth, support, and sensitivity are more inclined to foster a secure attachment pattern in the child (Bowlby, 1973). Moreover, those with more secure attachment patterns also tend to be more mindful (Shaver et al., 2007; Sharaf et al., 2009; Pepping et al., 2015; Melen et al., 2017). According to Ryan et al. (2007), this sense of security can help to create the foundation for the maturity of awareness as well as contextual attention. Conversely, difficulties developing mindfulness have been associated with the emergence of an insecure attachment style, which is facilitated by unresponsive or inconsistent relationships between parents and their children (Bowlby, 1973; Pepping et al., 2013; Stevenson et al., 2017).

The association between parenting style and children’s behavioral adjustment level has been shown to be mediated by children’s behavioral and attentional regulation abilities (Cheah et al., 2009). As such, there is value in exploring mindfulness as a potential competence of adolescents, since it appears that having a loving, autonomous, and communicative family environment during childhood facilitates the development of mindfulness (Ryan et al., 2007), and this development persists into adolescence (Galla et al., 2020). This raises the possibility that adolescents who experience more positive parenting possess stronger skills in mindfulness.

Mindfulness is described as a state of consciousness that is developed through paying attention to the present moment with a non-judgmental attitude (Kabat-Zinn, 2003). A substantial corpus of literature supports the notion that mindfulness promotes health by shielding individuals against the adverse impacts of stress (Creswell and Lindsay, 2014), as well as suicidal thoughts and behaviors (Bentley et al., 2017; Per et al., 2022). There is growing evidence that mindfulness-based interventions (MBIs) are sufficiently effective in enhancing positive emotions and reducing depression, suicidal ideation (Serpa et al., 2014), and aggression (Fix and Fix, 2013). For instance, Zhu et al. (2021) discovered that increasing mindfulness training was associated with low level of depression. Recently, a meta-analysis indicated that MBIs moderately reduced suicidal ideation (Schmelefske et al., 2020) and other psychological outcomes linked to suicide (e.g., depression; Witt et al., 2019). MBIs may also be effective in reducing aggression (Peters et al., 2015; Gillions et al., 2019). For instance, Heppner et al. (2008) found a negative correlation between mindfulness and self-reported aggressiveness, whereby increasing people’s mindfulness in an experimental setting resulted in less aggressive behavior. Similar to positive parenting, mindfulness has been shown to prospectively and robustly predict anxiety, depression, aggression, and social impairment (Williams, 2008; Fix and Fix, 2013; Bajaj et al., 2016; Westphal et al., 2021). Considering previous findings, it is plausible that mindfulness could mediate the link between positive parenting and maladaptive psychological outcomes such as aggression, depression, and suicidal ideation.

Although few studies have directly demonstrated the association between positive parenting and mindfulness, a recent study by Nieto-Casado et al. (2022) found that mindfulness partially mediated the association between parental competency and anxious-depressive symptoms and suicidal ideation. The authors speculated that this mediation effect was due to the fact that mindfulness inhibits the activation of dysfunctional thought patterns and maladaptive coping methods, which are common in individuals with anxious-depressive symptomatology. Based on the SEL model, positive parenting (i.e., the environment) may help individuals to develop skills in mindfulness (i.e., individual competence), which in turn reduce the likelihood of experiencing depression, aggression, and suicidal ideation. If so, it is reasonable to speculate that positive parenting could improve or cultivate mindfulness in adolescents, and, in turn, mindfulness could influence maladaptive psychological outcomes. Individual mindfulness could be promoted through the family environment, and thus decrease maladaptive psychological outcomes. However, considering the Western–Eastern cultural differences in parenting practices, further research into the associations between positive parenting and aggression, depression, and suicidal ideation in China is required. In addition, most studies into parenting style and adolescent maladaptive psychological outcomes have been cross-sectional, which cannot accurately reveal quasi-causal relationships between variables. The current study used a three-wave longitudinal method to explore the associations of positive parenting and mindfulness with maladaptive psychological outcomes in a sample of middle school adolescents.

**FIGURE 1:**
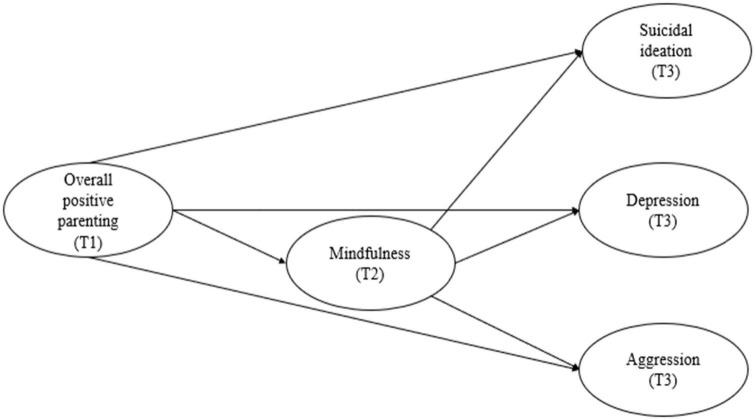
A hypothetical model of the effects of overall positive parenting and mindfulness on aggression, depression, and suicidal ideation.

We used a mediation model (Figure 1) to examine the intermediary effect of mindfulness on positive parenting and adolescent maladaptive psychological outcomes. Previous studies have found that socio-demographic factors (e.g., sex, age, household income, and education) are associated with suicidal ideation (Groleger et al., 2003; Grunbaum et al., 2004; Swahn and Bossarte, 2007; Kim et al., 2016; Cabello et al., 2020), depression (Mohammadi et al., 2019), and aggression (Stipek and Miles, 2008). To reduce endogeneity bias, we adjusted for the demographic variables of age, sex, and subjective social status. Based on the existing literature, the following hypotheses were made:

Hypothesis 1: Positive parenting positively predicts mindfulness.

Hypothesis 2: Positive parenting and mindfulness negatively predict adolescent suicidal ideation, depression, and aggression.

Hypothesis 3: The associations between positive parenting and suicidal ideation, depression, and aggression in adolescents are mediated by mindfulness.

## Materials and methods

### Participants

This study employed a convenience sampling method to recruit middle school students from two schools in Henan Province, China. A total of 386 students self-reported their perceived positive parenting at Time 1 (T1; October, 2021), 385 students self-reported their mindfulness at Time 2 (T2; December, 2021), and 375 students self-reported their depression, aggression, and suicidal ideation at Time 3 (T3; February, 2022). Sample attrition was *N* = 11. Due to school transfers or incomplete responses, the excluded individuals could not be followed longitudinally. Of the final 375 participants, their average age was 13.48 ± 0.88 years at T1; 192 (51.2%) were male and 183 (48.8%) were female; 105 (28%) were in grade 7 and 270 (72%) in grade 8; 34 (9.1%) were only children and 341 (90.9%) were not.

### Procedure

Signed informed consent was obtained from all participants prior to completion of the questionnaire, after approval had been granted by school principals. The students were informed that their participation would have no bearing on the educational options available to them in middle school, and that their participation was entirely voluntary. T1 data collection was performed in October 2021, when adolescents were in grades 7–8; data collection at T2 was in December 2021; and data collection at T3 was performed in February 2022, when students were still in the same grade as they were at T1.

### Measures

#### The positive parenting subscale (time 1)

Positive parenting was measured using the 14-item Emotional Warmth subscale from the Chinese version of the Egna Minnen Beträffande Uppfostran, developed by Perris et al. (1980) and validated by Li et al. (2012). Seven items assess the father’s positive parenting (e.g., “My father showed with words and gestures that he liked me”) and seven items assess the mother’s positive parenting (e.g., “I felt that my mother was proud when I succeeded in something I had undertaken”). All items were rated by participants on a 4-point scale ranging from 1 (almost never) to 4 (always), with higher overall scores indicating higher perceived positive parenting. In this investigation, the Cronbach’s α-value was 0.95.

#### The mindful attention and awareness scale (time 2)

Five questions from the Chinese Version of the Mindful Attention and Awareness Scale (Deng et al., 2012), which was created by Brown and Ryan (2003) and modified by Hülsheger et al. (2013), were used to assess mindfulness. A sample item is “I find myself doing things without paying attention.” Each item was scored on a Likert scale ranging from 1 (almost never) to 6. (always). Higher scores indicated greater mindfulness. In this investigation, the Cronbach’s α-value was 0.91.

#### The beck scale for suicidal ideation (time 3)

Adolescent suicidal ideation was measured using the Chinese version (Zhang and Brown, 2007) of the Beck Scale for Suicide Ideation (Beck and Steer, 1991). The scale consists of 19 self-report items, and participants are asked to rate each item on a 3-point scale. Higher scores indicate a greater tendency toward suicidal ideations. In this investigation, the Cronbach’s α-value was 0.90.

#### The patient health questionnaire (time 3)

Depression was measured using the Chinese version of the Patient Health Questionnaire (PHQ-9; Wang et al., 2014), which is a widely used instrument and consists of nine items. Participants rated each item on 4-point Likert scale ranging from 1 (not at all) to 4 (nearly every day), on the basis of how much a symptom had bothered them over the last 2 weeks. The PHQ-9 has very high levels of both reliability and construct validity (Kroenke et al., 2001). In this investigation, the Cronbach’s α-value was 0.89.

#### The Chinese version of the Buss–Perry aggression questionnaire (time 3)

The Chinese version of the Buss–Perry Aggression Questionnaire (BPAQ) is a valid and accurate indicator of adolescent aggression that is based on empirical research (Maxwell, 2007). This self-report scale assesses the following four different dimensions of aggressive behavior: physical aggression, verbal aggression, anger, and hostility. All items are assessed on a Likert scale ranging from 1 (not at all like me) to 5 (completely like me). Higher overall scores represent more aggression. In this investigation, the Cronbach’s α-value was 0.90.

#### Family socioeconomic status

Information on each participant’s family socioeconomic status (family SES; i.e., the annual family income, highest parental occupation, and highest educational level achieved by parents) was collected from adolescents using questions in line with the Program for International Student Assessment (OECD, 2012). The standard scores of these three variables were analyzed using principal component analysis, and the factor scores were saved as family SES scores. The family SES scores in this study ranged from −2.39 to 2.58, whereby higher scores indicated a higher family SES.

### Data analyses

In the first step, we assessed bivariate Pearson’s correlation coefficients for the key variables. In the second step, we performed structural equation modeling with the maximum likelihood estimator (Muthén and Muthén, 1998-2017) to test our hypothetical model (see Figure 1). Given that there was a strong association between positive parenting of the father and mother, the overall positive parenting was calculated by combining the positive parenting items into two parcels using a domain-representative technique to prevent exaggerated measurement errors due to many latent variable items (Little et al., 2002). Similarly, the BPAQ and PHQ-9 items were grouped into three and four parcels, respectively, using a domain-representative technique (Little et al., 2002). A full information maximum likelihood estimator was used to address the missing data issue, which is the suggested strategy for dealing with missing random data (Enders, 2010). Sex (male = 1), age, and family SES were entered in the structural equation models as covariates. The comparative fit index (CFI), Tucker-Lewis index (TLI), and root mean square error of approximation (RMSEA) were used to assess the adequacy of the model. CFI and TLI values greater than 0.95 and RMSEA values less than 0.06 indicate a good model fit, whereas CFI and TLI values greater than 0.9 and RMSEA values less than 0.08 indicate a satisfactory model fit (Hu and Bentler, 1999; Marsh et al., 2005). In the third stage, we employed bootstrap techniques with 5,000 samples and 95% confidence intervals to examine various mediation effects (Shrout and Bolger, 2002).

## Results

### Associations between study variables

The means and standard deviations of all variables, as well as the bivariate correlations between variables, are reported in Table 1. Overall positive parenting was positively correlated with mindfulness (*r* = 0.28, *p* < 0.001), and both overall positive parenting and mindfulness were negatively correlated with suicidal ideation, depression, and aggression (*r* = −0.35 to −0.16, all *p* < 0.01).

**TABLE 1:** Descriptive statistics and correlations between variables.

	*M*	*SD*	1	2	3	4	5
1. Overall positive parenting	2.71	0.80	0.95	0.28***	−0.28***	−0.20***	−0.16**
2. Mindfulness	4.14	1.34		0.91	−0.22***	−0.30***	−0.35***
3. Suicidal ideation	1.27	0.44			0.90	0.64***	0.44***
4. Depression	1.62	0.73				0.89	0.57***
5. Aggression	1.91	0.79					0.90

Coefficient alphas are listed in the diagonal of the table. **p < 0.01; ***p < 0.001.

### Pathway analysis

**FIGURE 2:**
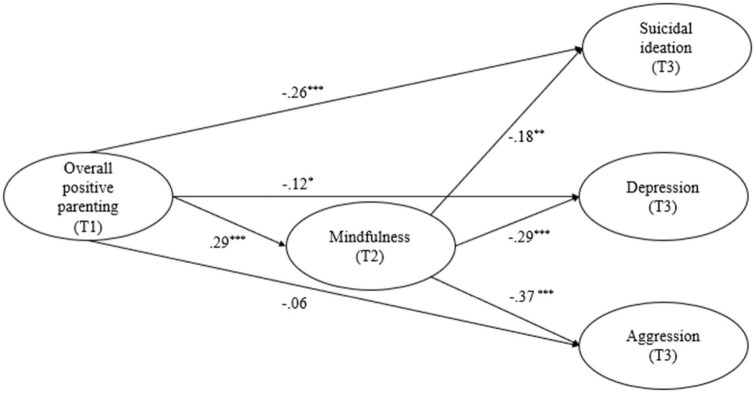
The longitudinal mediating role of mindfulness in the association between adolescent overall positive parenting, suicidal ideation, depression, and aggression, with standardized coefficients. Results of covariates (i.e., sex, age, and family SES) are not shown for the purpose of simplicity.

The hypothetical model showed a good fit to the data (CFI = 0.977, TLI = 0.973, RMSEA = 0.039, 90% confidence interval = [0.030, 0.047]), and all factor loadings of the latent variables were significant and above 0.60. Sex, age, and family SES did not predict suicidal ideation, depression, or aggression. Figure 2 and Table 2 present the direct and indirect effects. Overall positive parenting positively predicted mindfulness (β = 0.29, *p* < 0.001), negatively predicted suicidal ideation (β = −0.26, *p* < 0.001) and depression (β = −0.12, *p* = 0.03), and did not significantly predict aggression (β = −0.06, *p* = 0.314). Mindfulness negatively predicted suicidal ideation (β = −0.18, *p* = 0.004), depression (β = −0.29, *p* < 0.001), and aggression (β = −0.37, *p* < 0.001). All the indirect effects of overall positive parenting on suicidal ideation (β = −0.05, *p* = 0.012), depression (β = −0.08, *p* = 0.001), and aggression (β = −0.11, *p* < 0.001) *via* mindfulness were significant, as zeros were present in the bias-corrected bootstrap 95% confidence intervals.

**TABLE 2:** Results of direct and indirect effects of the hypothetical model.

Variable/Paths	β	*B*	*SE*	*p*	95% CI
**Direct effects**					
Overall positive parenting → Mindfulness	0.29	0.42	0.09	<0.001	0.19, 0.39
Overall positive parenting → Suicidal ideation	–0.26	–0.09	0.03	<0.001	−0.39, −0.12
Overall positive parenting → Depression	–0.12	–0.11	0.05	0.03	−0.23, −0.01
Overall positive parenting → Aggression	–0.06	–0.05	0.05	0.314	−0.17, 0.07
Mindfulness → suicidal ideation	–0.18	–0.04	0.02	0.004	−0.30, −0.06
Mindfulness → depression	–0.29	–0.17	0.04	<0.001	−0.40, −0.16
Mindfulness → aggression	–0.37	–0.20	0.04	<0.001	−0.49, −0.23
**Indirect effects**					
Overall positive parenting→ Mindfulness → Suicidal ideation	–0.05	–0.02	0.01	0.012	−0.10, −0.02
Overall positive parenting → Mindfulness → Depression	–0.08	–0.07	0.02	<0.001	−0.13, −0.04
Overall positive parenting→ Mindfulness → Aggression parenting	–0.11	–0.09	0.02	<0.001	−0.17, −0.06

β, standardized coefficient, B, non-standardized coefficient; SE, standard error; CI, confidence interval.

## Discussion

While numerous studies have identified negative associations between positive parenting and depression, aggression, and suicidal ideation in adolescents, we know relatively little about the role of mindfulness as a mechanism underlying these relationships, particularly in a Chinese context. Furthermore, previous studies have generally been cross-sectional, which makes it difficult to make causal inference. The current study fills this gap in the literature by investigating whether mindfulness mediates the relationships between positive parenting and depression, aggression, and suicidal ideation. This is also the first study to take a longitudinal perspective in exploring this in a Chinese context. Several important and novel findings were revealed. First, positive parenting was positively associated with mindfulness longitudinally. Second, positive parenting and mindfulness were both negatively correlated with depression, aggression, and suicidal ideation. Moreover, mindfulness measured at T2 partially mediated the relationship between positive parenting at T1 and depression and suicidal ideation at T3, and fully mediated the relationship between positive parenting at T1 and aggression at T3.

### Positive parenting positively predicts mindfulness

As the highlight of our study, positive parenting was positively correlated with mindfulness, as predicted in Hypothesis 1. To the best of our knowledge, this is the first investigation to show that positive parenting is correlated with mindfulness using a longitudinal study design. This finding is consistent with another study, which revealed that parental competency was positively associated with adolescent mindfulness (Nieto-Casado et al., 2022). Another study has suggested that the origins of mindful attention and awareness lie in relationships between caregivers and their children (Ryan et al., 2007). According to the family system theory (Bowen, 1974), positive parenting is a family environment resource that can support adolescents’ development of personal competences and help children to establish healthy attachment bonds with their caregivers. The family system theory highlights the significance of the family system for the development of mindfulness, and suggests that mindfulness may be influenced, at least in part, by family relationships and experiences (Bowen, 1974). For example, dispositional mindfulness was lowest among adolescents who reported frequent/intense interparental conflict and lowest-quality relationships with their mothers (Lucas-Thompson et al., 2021). In line with this, the experience of positive, supportive warmth from caregivers has been reported to create a sense of security that helps adolescents develop an attentiveness to their own experiences (Parker et al., 2015), which assists the development of a set of cognitive-emotional patterns that promote open, receptive, non-judgmental awareness and attentiveness to whatever circumstances may present themselves (Caldwell and Shaver, 2015). Recently, Lan (2022) reported that parental warmth is more likely to promote positive parent–child connections characterized by affectionate and cooperative interactions. Additionally, children who grow up in warm and supportive families are more likely to internalize their parents’ values, which in turn enhances their capacity to self-regulate (Carlo et al., 2011; Ngai et al., 2018). Moreover, positive parenting, which entails a balanced approach to offering warmth, respect, autonomy, communication, and support without disregarding acceptable degrees of control and regulation, has been found to increase children’s level of adjustment competence (Schofield et al., 2014). In other words, positive parenting facilitates the development of positive relationships with adolescents, which in turn helps to cultivate mindfulness abilities.

### Positive parenting and mindfulness negatively predict adolescent suicidal ideation and depression

The correlation results showed that positive parenting and mindfulness were both longitudinally negatively associated with depression and suicidal ideation, while only mindfulness was negatively associated with aggression longitudinally, which supports Hypothesis 2, as well as the results of earlier studies (Bamaca-Colbert et al., 2018; Zhu et al., 2021). Some studies have found that positive parenting is linked to a reduced risk of depression (Feng et al., 2009; Clayborne et al., 2021), suicidal ideation, and behavior (Lai and McBride-Chang, 2001), and aggressive behavior (Chen and Raine, 2018) in adolescents. A negative relationship between mindfulness and depression has also been reported in several studies (Christopher and Gilbert, 2010; Bajaj et al., 2016; Kircaburun et al., 2019; Al Harbi et al., 2021; Sharma and Kumra, 2022). Furthermore, a growing body of research has shown that MBIs can effectively reduce depression and aggression (Kriakous et al., 2021; Rezakhani and Vakili, 2021) in both clinical and non-clinical populations (Williams et al., 2000; Williams, 2008).

Nonetheless, our results do not fully support Hypothesis 2, in that we found no direct effect of positive parenting on adolescent aggression. However, this does not refute the notion that positive parenting affects the development of aggression. It is worth noting that previous studies on the relationship between positive parenting and aggression have produced mixed results, including non-significant results in cross-cultural studies. For example, in a juvenile sample of 11–12 year olds (51% boys, 80% African Americans), Chen and Raine (2018) found a negative relationship between positive parenting and aggression among those who matured early, but not among those who matured on time/late. However, another study presented different results, whereby positive parenting was unrelated to aggression in early maturing girls (mean age = 11.25 years; Mrug et al., 2008). Moreover, Yaman et al. (2010) found no main effect of positive parenting on the prediction of toddler aggression. As suggested by meta-analysis findings from Rothbaum and Weisz (1994), the relationship between parenting quality and child aggression may be weaker in toddlers and preschoolers than in older children. In addition, a separate meta-analysis found that the association between positive parenting and reduced aggression was strongest among college students, moderate among seniors, and minimal among middle and elementary school students in China (Lei et al., 2018). The reasons for these contradictory findings could lie in the use of different instruments for measuring outcomes, or in the demographic makeup of the study population (Lei et al., 2018).

There are various explanations for the present result of no direct effect of positive parenting on adolescent aggression. As opposed to depression and suicidal ideation, the aggression we measured, which includes physical aggression, verbal aggression, anger, and hostility, was related to interpersonal interactions. First, the effect size of correlations between positive parenting and aggression was small (*r* = −0.16, *p* < 0.01). Given that mindfulness mediated the relationship between positive parenting and aggression, positive parenting had no direct influence on adolescent aggression. Second, there may be some factors (peer friendships, the environment, etc.) that account for the indirect relationship between positive parenting and aggression. For instance, Hartup and Stevens (1997) posited that the influence that parents exert over children’s behavior decreases as peer relationships and friendships grow more salient and relationships with parents become less important on entering adolescence. Third, individual characteristics (such as emotional regulation or hostile attribution biases) could influence the indirect relationship between positive parenting and aggression (Kawabata et al., 2011). This result may also be explained by the idea that positive parenting promotes socio-cognitive traits such as emotion management, self-confidence, and social competence, all of which can help to prevent the emergence and maintenance of aggression (Kawabata et al., 2011). For instance, previous studies have indicated that children who are exposed to positive parenting are provided with a socializing situation that promotes their ability to regulate emotions and social competence, both of which may be linked to a lower incidence of aggressive behavior (Parke et al., 1992; Zhou et al., 2002).

### Adolescent mindfulness mediates the relationship between positive parenting and suicidal ideation, depression, and aggression

We found that mindfulness mediated the effect of positive parenting on adolescent depression, aggression, and suicidal ideation, which supports Hypothesis 3. More specifically, the mediation effect was only partial due to the direct effects of positive parenting on depression and suicidal ideation in the structural model. However, mindfulness completely mediated the association between positive parenting and aggression. Mindfulness can be defined as an awareness of our thoughts, feelings, physical sensations, and surrounding environment on a moment-to-moment basis. Being mindful is associated with openness, non-judgment, awareness, friendliness, acceptance, compassion, and kindness (Baer et al., 2019), and can reduce rumination and the frequency of both depressive symptoms (Moreira and Canavarro, 2018) and suicidal outcomes (Hargus et al., 2010; Nieto-Casado et al., 2022). Recent work has demonstrated that mindfulness partially mediates the correlations between parental competence and both anxious-depressive symptoms and suicidal ideation in adolescents (Nieto-Casado et al., 2022). The SEL model can be used to explain the role of mindfulness as a mediator in the association between positive parenting and adolescent depression, aggression, and suicidal ideation identified in this study (Tolan et al., 2016). During parent–child interactions, a supportive, encouraging, and warm parent is more likely to model sensitivity and attentiveness to the adolescent, which can create a safe and supportive environment that conveys to the adolescent that they are loved and accepted (World Health Organization [WHO], 2007; Juffer et al., 2012). Adolescents who feel that their parents accept them are more inclined to interact with the world in a more mindful way. Mindfulness can foster a more profound and long-lasting awareness of being engaged with whatever is occurring in the present moment, with a recognition that every moment, whether positive or negative, is temporary and will be promptly replaced by a new experience (Kabat-Zinn, 2003). To put it another way, mindfulness promotes greater acceptance and less reactivity to whatever is taking place on physical, cognitive, affective, and behavioral levels, and also increases flexibility and accuracy in one’s perception about what is taking place in the present moment (Duncan et al., 2009). From this perspective, adolescents who have higher levels of mindfulness are better able to remain open and responsive to their current negative experiences rather than judging, ignoring, or minimizing them, which in turn makes them less prone to suffer depression or suicidal ideation (Kabat-Zinn, 2003; Wallace and Shapiro, 2006; Coffey et al., 2010).

The current study also found an indirect association between positive parenting and aggression, which was fully mediated by mindfulness in adolescents. A key aspect of mindfulness is being aware of one’s own impulses and realizing that they are transient, which strengthens an individual’s ability to resist action until the impulse has passed (Katz and Toner, 2013). This means that adolescents with high levels of mindfulness are more likely to have a deeper comprehension of their cognitive and behavioral processes, and are able to learn to respond more intentionally and consciously rather than reactively or automatically (Kabat-Zinn, 2003). In this case, flexibility and awareness can help them see other possibilities or options, rather than seeing aggression as the only viable response. Some studies have painted a negative picture of Chinese parenting, characterizing it as overbearing and even hostile (Chao, 1994). However, adolescents living in families with positive parenting may learn to regulate their negative emotions in adaptive ways by observing their parents’ behavior. This can result in more psychosocial adjustment, which may decrease the likelihood of mental health problems.

These findings are largely congruent with SEL theory (Tolan et al., 2016), which argues that parents can have long-lasting positive impacts on their children. Namely, adolescents cumulatively learn more self-awareness, self-management, and relationship skills from their parents’ positive support, which they are able to put into practice as they gain more freedom to act. We speculated that when children feel that they are cared for, encouraged, supported, and accepted through their parents’ behavioral style, they foster the capacity to accept themselves and their own emotional state. In other words, positive parenting may help to cultivate mindfulness in adolescents, and this mindfulness could mitigate depression, aggression, and suicidal ideation.

### Implications and recommendations

First, evidence from this study bolsters the public health emphasis on improving and encouraging positive parenting skills for better adolescent mental health. The present findings suggest that it is necessary to develop a positive parent–child relationship to foster positive adolescent development. Hence, schools or governments could offer education or training that provides parents with information about positive parenting, as well as the skills they need to parent effectively, which would promote positive development and reduce negative outcomes for youths. The second important point is that our model’s emphasis on the importance of mindfulness suggests that positive parenting practices can cultivate mindfulness, which would result in greater improvement in mental health. Moreover, whether the parenting practice is positive or not, mindfulness interventions could improve the consequences of parenting practice.

### Limitations and future research

Despite the numerous strengths of this longitudinal study, some limitations must be noted. First, all of the data came from participants’ subjective reports; future studies should incorporate other forms of evidence (such as reports from teachers and classmates) to bolster the reliability of our results. Future studies could also overcome this limitation by using multi-source data collection methods or experimental method designs to test our theoretical models and determine the causal relationships between variables. Second, participants were Chinese middle school students, so our results are not generalizable to Chinese adults. Third, while this research examined the mediation role of mindfulness in the link between positive parenting and maladaptive psychological outcomes, individual factors, such as adolescent resilience (Kaniušonytė and Laursen, 2022), may also mediate the effects of positive parenting on adolescent mental health. Therefore, future studies should focus on additional protective factors that might also play a mediating role in the link between positive parenting and adolescent mental health. Fourth, despite our use of longitudinal data, the time interval between the measurement waves was only 2 months. This may not be sufficient to detect the wave-to-wave influence of the constructs on one another. Future studies should explore the influence of positive parenting and mindfulness in multiple waves at longer time intervals to clarify how mindfulness mediates the effects of positive parenting and maladaptive psychological outcomes. Fifth, a longitudinal design enabled us to test mediation effects in a more rigorous manner than for cross-sectional designs. However, each variable was measured only once, and so our results could lack stability (Cole and Maxwell, 2003). Future studies should collect all variables at all waves and use a three-wave cross-lagged analysis to verify the model proposed in this study. Intervention studies investigating whether mindfulness changes aggression/depression/suicidal ideation in adolescents would also clarify the issue of directionality. In future research, we will use intervention or experimental methods to directly explore the relationship between independent variables (mindfulness) and dependent variables (depression, aggression, and suicidal ideation).

## Conclusion

This is the first study to examine the longitudinal relationship between positive parenting and mindfulness, aggression, depression, and suicidal ideation in a sample of Chinese middle school students. The current study expands on previous work and provides important insights that enhance our understanding of these relationships. Given that positive parenting may increase adolescents’ level of mindfulness, this mechanism could facilitate the design of depression and suicide intervention and prevention programs.

## Data availability statement

The data analyzed in this study is subject to the following licenses/restrictions: The datasets used and/or analyzed during the current study are available from the corresponding authors on reasonable request. Requests to access these datasets should be directed to corresponding authors.

## Ethics statement

This study had been approved by the academic committee of Zunyi Medical University. Written informed consent to participate in this study was provided by the participants or their legal guardian/next of kin.

## Author contributions

YS, WS, and ZL designed the study. WS and ZL collected the data. HT, ZL, QZ, and GL analyzed and interpreted the data. YS drafted the manuscript. LH, YG, HT, and ZL revised the manuscript critically for important intellectual content. All authors agreed to be accountable.
